# Methods to measure calcitonin receptor activity, up-regulated in cell stress, apoptosis and autophagy

**DOI:** 10.12688/f1000research.72845.1

**Published:** 2021-10-07

**Authors:** Peter Wookey, Pragya Gupta, Lucas Bittencourt, Shane Cheung, David Hare, Sebastian Furness

**Affiliations:** 1 Medicine, University of Melbourne, Heidelberg, Victoria, 3084, Australia; 2 BOMP, University of Melbourne, Parkville, Victoria, 3052, Australia; 3 Drug Discovery Biology and Department of Pharmacology, Monash University, Parkville, Victoria, 3052, Australia; 4 School of Biomedical Sciences, Faculty of Medicine, The University of Queensland, St Lucia, Queensland, 4067, Australia

**Keywords:** cell stress, autophagy, apoptosis, calcitonin receptor isoform, antibody, antibody conjugate, high content screening, quantitative PCR, nanopore sequencing

## Abstract

The expression of the calcitonin receptor (CT Receptor) is widespread throughout the life cycle of mammals and in many diseases, and in these contexts the functions of the common isoforms is largely unknown. The relatively recent development of anti-CT Receptor antibodies that bind separate epitopes on the CT _a_ Receptor and CT _b_ Receptor isoforms has advanced our knowledge and understanding of these events. CT Receptor at the protein level is upregulated in programmed cell death including apoptosis (as described in a previous publication) and autophagy, which is discussed in our upcoming, unpublished review. Incomplete data sets are cited in this review on the upregulation of CACLR (encoding CT Receptor) mRNA, in particular the insert-positive isoform (CT _b_ Receptor), in response to cell stress. Cell stress is induced by growth in depleted foetal bovine serum (dFBS) or without FBS, both of which induce degrees of starvation and autophagy, or dFBS plus staurosporine, which induces apoptosis. Details of the methods deployed to generate these data are described here including measurement of the upregulation of CT _b_ Receptor mRNA with qPCR and nanopore long range sequencing. An anti-CT Receptor antibody also known as CalRexin ^TM^, which binds an epitope in the N-terminal domain, was conjugated to either fluorophore 568, which is accumulated into apoptotic cells as previously reported, or pHrodo Red, a pH dependent fluorescent dye, which is accumulated into autophagic and apoptotic cells.  These conjugates are under development to image programmed cell death. The methods for conjugation and high content imaging on the Operetta platform are described. The high fluorescence intensity at low pH of CalRexin:pHrodo Red in both autophagic and apoptotic cells suggests localisation in autophago-lysosomes and lysosomes respectively. Overall, these observations and the methods that underpin them have contributed to our understanding of the widespread expression of CT Receptor isoforms.

## Introduction

**Figure 1.**
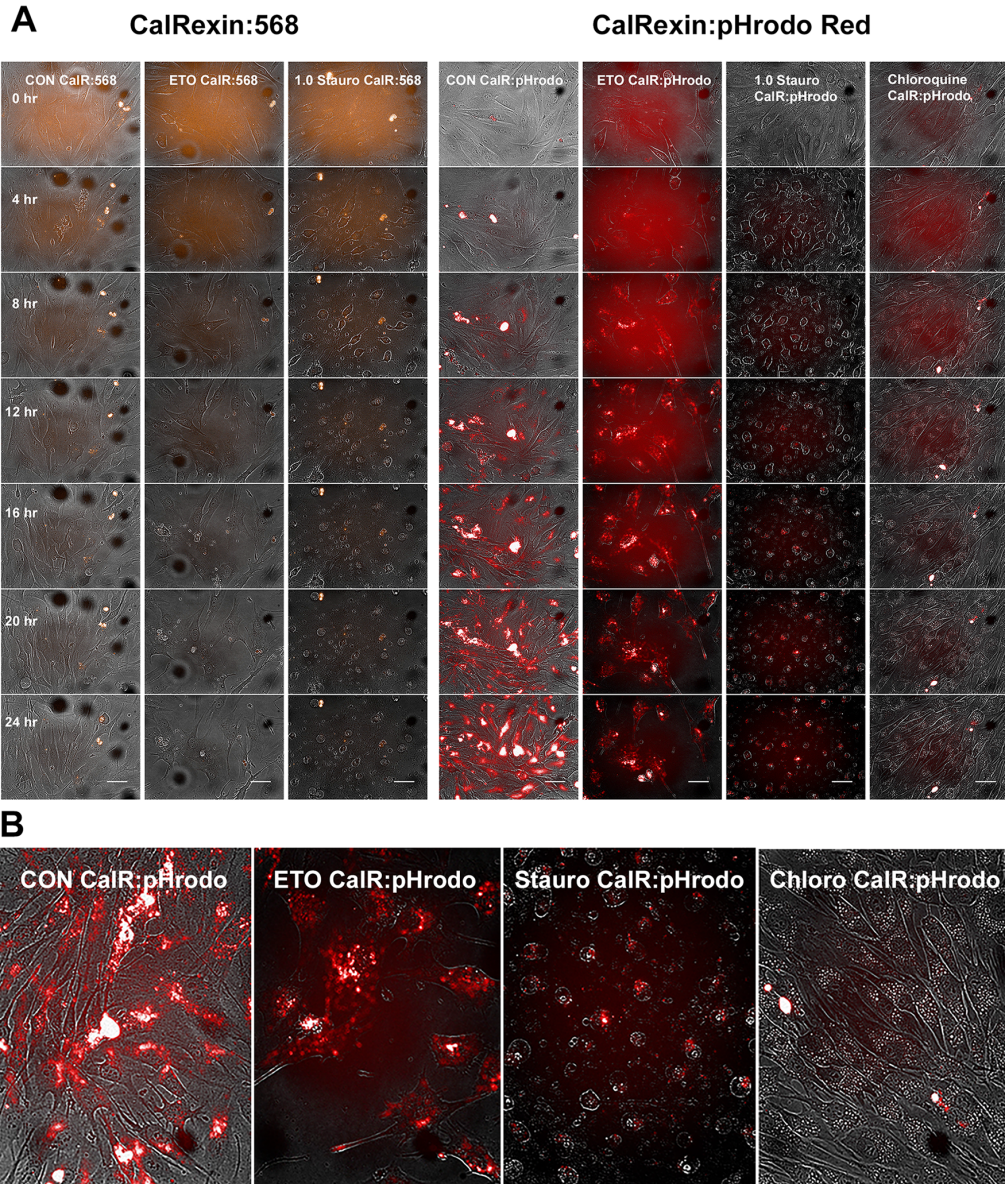
Imaging autophagy and apoptosis in MG63 in real time cells on the Operetta platform. (A) CalRexin ^TM^:pHrodo Red compared to CalRexinTM:568 for imaging MG63 cells grown on Matrigel and undergoing autophagy (control [CON] starved by growth in depleted serum), or to induce apoptosis treated with 50 μM etoposide or 1 μM staurosporine, or treated with 50 μM chloroquine to inhibit formation of autophagolysosomes. ^4^ Images were recorded every 30 minutes on the Operetta platform (Images displayed above were captured at 0, 4, 8, 12, 16, 20 & 24 hours). The scale bar in the 24-hour images is 50 μm. (B) Enlarged images recorded at 20 hours. Note that chloroquine known to inhibit the fusion of autophagosomes with lysosomes to form autophago-lysosomes inhibits fluorescence with CalRexin:pHrodo Red.

This article provides details of the methods developed to generate unpublished data, which is cited in an unpublished review article due for submission. The purpose is to provide the opportunity for researchers to review the methods, and if motivated, reproduce (and extend) the data. A panel of the unpublished data presented in the review is also include as Figure 1 below.

The methods used to generate unpublished data cited in an upcoming review, relate to the experiments with qPCR and nanopore sequencing in which the mRNA of the CT _b_ Receptor (insert-positive isoform, exon 10 spliced in), which together demonstrate upregulation in stressed cells. These methods also include synthesis of the novel imaging reagents CalRexin ^TM^:568 ( **RRID:AB_2893120**) and CalRexin ^TM^:pHrodo Red for imaging apoptosis and autophagy. All the data generated with these methods will be published in full in an experimental peer-reviewed manuscript that is currently being drafted and completed for publication.

## Methods

### Antibody production

The mouse monoclonal antibody mAb2C4 (clone 46/08-2C4-2-2-4, CalRexin ^TM^ (RRID:AB_2893093) was developed using standard techniques summarised below for the cloning of high expression hybridomas. CalRexin ^TM^ binds in the N-terminal domain of the CT Receptor isoforms, CT _a_ or CT _b_ Receptor. Data supporting the validation of the antibody CalRexin ^TM^ have been presented in the supplementary materials linked to. ^1^

The strategy to develop CalRexin ^TM^ is as follows. A peptide equivalent to the epitope in the N-terminal domain of the human CT receptor (C-PSEKVTKYCDEKGVWFK-NH2) was synthesized ( Auspep, Tullamarine, Australia). This peptide was conjugated to Keyhole Limpet Hemocyanin (KLH) via a linker maleimidocaproyl-N-hydroxysuccininmide (Auspep, Australia). The rest of the procedure was performed by the dedicated laboratories at the Antibody Facility of the Walter & Eliza Hall Institute, Bundoora, Victoria, Australia. At the facilities of this third party provider, four mice were each immunized with the KLH conjugate essentially as described previously. ^2^ The spleens from the mice were removed on the day of fusion and a single cell suspension was prepared. The cells were then washed three times in Dulbecco’s modified Eagle’s medium (DMEM). Cells of the myeloma line (SP2/O) were washed three times in DMEM and adjusted to 0.5-1.0 × 10 ^8^ cells per fusion with a mouse spleen. Spleen and myeloma cells were mixed, pelleted and the fusion performed by addition of 1 mL warm polyethylene glycol 1500 to the pellet with gentle stirring (1 min). The fusion mixture was then added slowly to 25 mL DMEM. Cells were centrifuged and resuspended in hybridoma serum-free medium containing 10 % foetal bovine serum (FBS), interleukin-6 conditioned medium and HAT (hypoxanthine, aminopterin, thymidine), and plated out in 5 to 6 microtiter plates.

After incubation of the cells for 8 to 9 days, supernatants were collected at day 14 and tested by enzyme-linked immunosorbent assay (ELISA), checked by immunohistochemistry, before positive cells were selected for limiting-dilution cloning. Usually two rounds of cloning were sufficient to ensure the hybridomas were clonal.

Cloned cells with the desired isotype and exhibiting a high titre by ELISA test were grown in bioreactors. The monoclonal antibody was partially purified by Protein A chromatography (in mouse tonicity PBS). The selected clone (46/08-2C4-2-2-4) was used to produce the anti-human CT Receptor monoclonal antibody [isotype IgG1, kappa] adjusted to a final concentration of 1 mg/mL protein (sterile filtered) and stored at −80°C until required. Freeze/thaw cycles were avoided.

### Conjugations of antibody CalRexin ^TM^

The method of conjugation for CalRexin (mAb 2C4) and AF568:NHS esters to synthesise CalRexin:568 (RRID:AB_2893120) has been described previously. ^1^ The molar ratio of antibody to dye was 1:15 and in the case of CalrexinTM:pHrodo Red (RRID:_2893121) 15 mg of antibody was conjugated with 1 mg of pHrodo Red NHS esters (Thermo Fisher Scientific, TFS).

The antibody was desalted by centrifugation using 5 mL Zeba column (TFS) and the buffer replaced with 100mM sodium bicarbonate, pH 8.3. The dye pHrodo Red NHS esters (MW 650, 1 mg, 1.54 μmoles) was dissolved in 150 μL of anhydrous DMSO and added dropwise to the antibody in bicarbonate buffer. After 1 hr gentle inversion at RT the reaction was quenched with 50 μL of 1.5 M triethanolamine (20% solution in bicarbonate buffer) for 3 hr.

The conjugate can be separated from unreacted and other small molecules on a Zeba column and the buffer exchanged for PBS.

### PerkinElmer Operetta high-content imaging system

The results of these data are included in Figure 1. MG63 cells were cultured in MEMα (Thermo Fisher Scientific, TFS) plus 10% foetal bovine serum (TFS), washed once in MEMα plus 10% depleted FBS (dFBS) and plated at 10,000 cells per well in a 96-well plate (CellCarrier-96 Ultra, PerkinElmer #6055302) in MEMα/10% dFBS.

To make dFBS, FBS centrifuged at 100,000 × g for 24 hours and the clear supernatant, devoid of exosomes, was collected as depleted FBS (dFBS) and the pellet discarded.

CalRexin ^TM^:AF568 (2 μg/mL, Apop Biosciences Pty Ltd) or CalRexin ^TM^:pHrodo Red (2 μg/mL, Apop Biosciences Pty Ltd) was added to the appropriate wells of the 96-well plate. Staurosporine (1 μM final concentration, Sigma), etoposide (50 μM final concentration, Sigma) or chloroquine (50 μM) were added at time zero minutes.

Cells were imaged on an Operetta high-content screening system ( Operetta CLS, RRID:SCR_018810) controlled by the Harmony software (Harmony version 4.1, RRID:SCR_018809). Cells were imaged over a 24 h period with images being acquired at a single focal plane every 30 min using a 40× high NA (Numerical Aperture) objective. The system was maintained at a constant temperature of 37 °C and 5% CO _2_ for optimal cell growth.

The following filter sets were used for imaging each dye: for CalRexin:pHrodo excitation was 520-550 nm and emission was 560-630 nm, and for CalRexin:AF568 excitation was 560-580 nm and emission was 590-640 nm. Excitation was provided by a Xenon lamp and transmission was set to 50 %. The brightfield channel was acquired with an exposure of 100 ms.

Time-lapse videos were created and annotated using FIJI image analysis software, version 1.53c ( Fiji, RRID:SCR_002285).

### RNA extraction

RNA was extracted using RNeasy Mini Kit ( Qiagen). The purity and concentration of the RNA was checked on a Nanodrop (A260/A280 ratio). To check RNA integrity 100 ng of RNA from each sample was electrophoresed on a 1.2 % Agarose gel and visualized using GelGreen ( Fisher Biotec) on ChemiDoc MP Imaging system ( Bio Rad ChemiDoc MP Imaging System, RRID:SCR_019037).

### RT-qPCR

Equal amounts of RNA from all the samples were used to synthesise cDNA using SuperScript VILO IV reverse transcriptase kit ( Invitrogen). qPCR assay targeting exon 10 of human CALCR mRNA (CT Receptor _b_ mRNA) was purchased from Thermo Fisher (Assay ID: Hs01016888_m1) along with GAPDH assay (Assay ID: Hs02786624_g1) as the housekeeping gene control. 20 μL duplex reaction was set up in a 96-well 0.1 mL qPCR microplate ( Applied Biosystems) by mixing 20 ng of cDNA, 1 μL of CALCR assay, 0.5 μL of GAPDH assay, 10 μL of 2X TaqMan™ Fast Advanced Master Mix (Applied Biosystems) and nuclease-free water, keeping three replicates for each sample. qPCR run was conducted for 50 cycles of 95°C for 1 sec and 60°C for 10 secs on QuantStudio™ 3 ( Applied Biosystems QuantStudio 12K Flex RealTime PCR System, RRID:SCR_021098). mRNA expression was quantified by calculating 2-∆∆Ct using Design and Analysis Software v2.5.1 ( Thermo Fisher).

### cDNA synthesis and amplification for nanopore sequencing

Primers specific to CT Receptor transcript 2 (NM_001742.3) were designed using SnapGene Viewer v5.0.4 ( SnapGene, RRID:SCR_015052) covering the full coding sequence including most of 3′ UTR. The forward primer is specific to the sequence corresponding to the exon 2 and exon 5 boundary and the reverse primer is specific to a 3′ UTR region in exon 17. Additionally, both primers have a universal barcode recognizing sequence (written in lower case, Oxford Nanopore Technologies). Forward primer was 5′tttctgttggtgctgatattgcGTGACAGAATTCCAGGACAAAGAGATC3′ and reverse primer was 5′acttgcctgtcgctctatcttcGGGCAGAACTATGTGCAATTCTATAATAAGC3′. cDNA synthesis was carried out using GoScript reverse transcriptase (Promega). 100-300 ng of cDNA was used for PCR amplification conducted for 25 cycles of 94°C for 30 sec, 60°C for 1 min, 65°C for 3 min using LongAmp Taq polymerase and final extension at 65°C for 10 min in a 25 μL reaction.

### Sample preparation and quality control for nanopore sequencing ^3^

PCR products were purified using 0.4× Agencourt AMPure XP ( Beckman Coulter) magnetic beads to remove short DNA fragments and the DNA concentration was measured by a Qubit 4 Fluorimeter ( Thermo Fisher Qubit fluorimeter, RRID:SCR_018095). To confirm the product length, 1 μL of each DNA sample was analysed on 4200 Tapestation system ( Agilent 4200 TapeStation System, RRID:SCR_019398) against D5000 DNA ladder (Agilent). DNA bands were visualised using Tapestation Controller Software v3.1.1 (Agilent). Samples having a band near 3.4 kb were selected for barcoding.

### Barcoding samples for nanopore sequencing

Each DNA sample (2 fmol) was barcoded using primers from PCR Barcoding kit ( EXP-PBC001) and PrimeStar GXL DNA Polymerase (Takara). The amplification was conducted for 12 cycles of 95°C for 15 sec, 62°C for 15 sec and 65°C for 3 min. Barcoded DNA was quantified using a Qubit 4 Fluorometer (Thermo Fisher) and purified using 0.4× Agencourt AMPure XP beads. Barcoded DNA, 15 fmol from each condition, were pooled together to make a DNA library using the 1D Nanopore Ligation Sequencing Kit (SQK-LSK109, Oxford Nanopore Technologies). DNA end repair and dA-tailing were performed using NEBNext Ultra II End-Repair/dA-tailing Module ( NEB, UK) for 5 min at 20°C and 5 min at 65°C. The DNA library was quantified using Qubit and purified using 0.6× Agencourt AMPure XP beads. 50 ng (or approximately 24.19 fmol for an estimated length of 3.4 kb DNA) of the DNA library was loaded on the MinION R9.4.1 flow cell (Oxford Nanopore Technologies). In total 1100 pores were active at the start of the run.

### Bioinformatic analysis using nanoPlot and TAQLoRe v1

Reads from the Nanopore sequencer, also known as 2D pass reads (basecall score ≥ 7) were analysed for quality and size distribution using NanoPlot v1.32.0 bioinformatic tool.

TAQLoRe v1 (Transcript Annotation and Quantification using Long Reads) pipeline ( https://github.com/twrzes/TAQLoRe) was used to create a CALCR hypothetical meta-gene by combining known and novel exons. Novel exons were defined as insertions (≥9 nucleotides) at a minimum distance of 6 nucleotides from known exons and having a minimum alignment proportion of 0.8. TAQLoRe then mapped the pass reads to the *CALCR* meta-gene as well as the human transcriptome. Mapped exons were annotated according to their location listed in the mapping output file. The only variant identified using this method was inclusion of exon 10. The abundance of exon 10 (%) was calculated for each staurosporine treatment vs. time sample (number of reads/total reads) to observe changes over time relative to the untreated control condition.

### Limitations of the data set

The limited data in Figure 1 will become part of a larger study to be published in a further experimental manuscript.

## Data availability

All data underlying the results are available as part of the article and no additional source data are required.
