# Supplementary material for: Methods to measure calcitonin receptor activity, up-regulated in cell stress, apoptosis and autophagy
Source: F1000Res. 2021 Oct 7;10:1019. [Version 1] doi: 10.12688/f1000research.72845.1 (PMC8543166; doi:10.12688/f1000research.72845.1)
Supplement: Peer review history

# Peer review: Reviewer response for version 1

**DOI:** 10.5256/f1000research.76452.r96415

**Published:** 2021-10-22

**Authors:** Eshan Ghosh

**Affiliations:** 1 Department of Drug Design and Pharmacology, Copenhagen University, Copenhagen, Denmark

The authors have provided a streamlined workflow to assess the expression of calcitonin receptor isoforms under stressed cell conditions. Authors have used three different approaches to exhibit differential receptor expression. The partial data for calcitonin receptor localization shows good use of CalRexin antibody conjugates for interrogating the receptor trafficking dynamics under specific conditions. Owing to the unavailability of data due to specific reasons given by authors, it is hard to appreciate all three approaches. However, the detailed method provided herein is sufficient for the scientific community to replicate the results. I would also like to request the authors to detail the conditions in which the RNA has been extracted so the exact conditions can be replicated.

Is the rationale for developing the new method (or application) clearly explained?

Yes

Is the description of the method technically sound?

Yes

Are the conclusions about the method and its performance adequately supported by the findings presented in the article?

Partly

If any results are presented, are all the source data underlying the results available to ensure full reproducibility?

Partly

Are sufficient details provided to allow replication of the method development and its use by others?

Yes

Reviewer Expertise:

cell biology, biochemistry, biophysics

I confirm that I have read this submission and believe that I have an appropriate level of expertise to confirm that it is of an acceptable scientific standard.

# Peer review: Reviewer response for version 1

**DOI:** 10.5256/f1000research.76452.r96413

**Published:** 2021-10-21

**Authors:** Virender Singh

**Affiliations:** 1 Department of Physiology and Biophysics, Case Western Reserve University, Cleveland, OH, USA

The article “Methods to measure calcitonin receptor activity, up-regulated in cell stress, apoptosis and autophagy” by Peter Wookey *et al.* describe methods to assess cell stress in terms of upregulation of CT receptor isoform(s) at mRNA level. The authors cover the generation and validation of anti-human CT Receptor antibody and CalRexin, later deployed to study cell stress. The authors have provided a clear context of why they are using CT Receptor as a readout of cell stress. The authors clearly described the technical details of the methods. For antibody production, the authors have used an epitope on the N-terminus, which is rational given that the N-terminus of a GPCR remains accessible to antibodies raised against; hence, it can be used on live cells. The authors have also compared different fluorophores, which confirms the flexibility of the antibody's use. Other researchers might want to use a fluorophore of their interest. The use of dFBS is not apparent. However, it is best to avoid exosomes containing serum while studying the uptake of compounds, from my understanding. The nanopore sequencing has been described well enough to follow. The authors have mentioned all minute details so that others can use the methods for their purpose. The authors have shown precise results of the techniques presented, except the nanopore data, which seems confidential at this stage.

 This is a fascinating and timely method paper, and I have thoroughly enjoyed reading and reviewing this article. The topic is of high importance. I am rather enthusiastic about this paper and supportive of its publication. I only offer minor suggestions to improve readability and enhance the paper's message (adopting them is optional).

**Minor issue:**

1. Scale bar for Figure 1B missing. 
2. An illustration summarizing the method in the form of a flow chart with checkpoints will be helpful for readers to visualize and understand the importance of work.

Is the rationale for developing the new method (or application) clearly explained?

Yes

Is the description of the method technically sound?

Yes

Are the conclusions about the method and its performance adequately supported by the findings presented in the article?

Yes

If any results are presented, are all the source data underlying the results available to ensure full reproducibility?

Yes

Are sufficient details provided to allow replication of the method development and its use by others?

Yes

Reviewer Expertise:

Neuroscience and cancer biology

I confirm that I have read this submission and believe that I have an appropriate level of expertise to confirm that it is of an acceptable scientific standard.
